# RETRACTION: Chitosan‐Coated Solid Lipid Nano‐Encapsulation Improves the Therapeutic Antiairway Inflammation Effect of Berberine against COPD in Cigarette Smoke‐Exposed Rats

**DOI:** 10.1155/carj/9758217

**Published:** 2026-02-19

**Authors:** Canadian Respiratory Journal

RETRACTION: H. Liu, Y. Li, X. Zhang, M. Shi, D. Li, and Y. Wang, “Chitosan‐Coated Solid Lipid Nano‐Encapsulation Improves the Therapeutic Antiairway Inflammation Effect of Berberine against COPD in Cigarette Smoke‐Exposed Rats,” *Canadian Respiratory Journal*, 2022, 8509396, https://doi.org/10.1155/2022/8509396.

The above article, published online on 15 April 2022 in Wiley Online Library (https://wileyonlinelibrary.com), has been retracted by agreement between the journal Editor‐in‐Chief, Prof. Alice Turner, and John Wiley & Sons Ltd. The retraction has been agreed due to image concerns related to Figure 1c being raised, both directly and on PubPeer [1]. Specifically, there are multiple instances where the lipid nanoparticles appear to be duplicated within Figure 1c, for which the authors were unresponsive when asked for an explanation.

The authors did not respond to the retraction.
